# Generation of Silver Standard Concept Annotations from Biomedical Texts with Special Relevance to Phenotypes

**DOI:** 10.1371/journal.pone.0116040

**Published:** 2015-01-21

**Authors:** Anika Oellrich, Nigel Collier, Damian Smedley, Tudor Groza

**Affiliations:** 1 Wellcome Trust Sanger Institute, Wellcome Trust Genome Campus, Hinxton, CB10 1SA, United Kingdom; 2 EMBL European Bioinformatics Institute, Wellcome Trust Genome Campus, Hinxton, CB10 1SD, United Kingdom; 3 National Institute of Informatics, Tokyo 101-8430, Japan; 4 School of ITEE, The University of Queensland, St. Lucia, QLD 4072, Australia; 5 Garvan Institute of Medical Research, 384 Victoria Street, Darlinghurst, NSW 2010, Australia; Bangladesh University of Engineering and Technology, BANGLADESH

## Abstract

Electronic health records and scientific articles possess differing linguistic characteristics that may impact the performance of natural language processing tools developed for one or the other. In this paper, we investigate the performance of four extant concept recognition tools: the clinical Text Analysis and Knowledge Extraction System (cTAKES), the National Center for Biomedical Ontology (NCBO) Annotator, the Biomedical Concept Annotation System (BeCAS) and MetaMap. Each of the four concept recognition systems is applied to four different corpora: the i2b2 corpus of clinical documents, a PubMed corpus of Medline abstracts, a clinical trails corpus and the ShARe/CLEF corpus. In addition, we assess the individual system performances with respect to one gold standard annotation set, available for the ShARe/CLEF corpus. Furthermore, we built a silver standard annotation set from the individual systems’ output and assess the quality as well as the contribution of individual systems to the quality of the silver standard. Our results demonstrate that mainly the NCBO annotator and cTAKES contribute to the silver standard corpora (F1-measures in the range of 21% to 74%) and their quality (best F1-measure of 33%), independent from the type of text investigated. While BeCAS and MetaMap can contribute to the precision of silver standard annotations (precision of up to 42%), the F1-measure drops when combined with NCBO Annotator and cTAKES due to a low recall. In conclusion, the performances of individual systems need to be improved independently from the text types, and the leveraging strategies to best take advantage of individual systems’ annotations need to be revised. The textual content of the PubMed corpus, accession numbers for the clinical trials corpus, and assigned annotations of the four concept recognition systems as well as the generated silver standard annotation sets are available from http://purl.org/phenotype/resources. The textual content of the ShARe/CLEF (https://sites.google.com/site/shareclefehealth/data) and i2b2 (https://i2b2.org/NLP/DataSets/) corpora needs to be requested with the individual corpus providers.

## Introduction

Despite improvements in technology, the underlying molecular mechanisms for approximately half of the recognised human genetic disorders, are unknown [1]. One of the consequences is that treatment and, more importantly, prevention opportunities are lacking. Furthermore, genetic disorders change their characteristics over time, causing the signs and symptoms to alter [2] and necessitating different treatments than in earlier stages. To allow for the identification of potential prevention and intervention mechanisms, the origin of a disease and its influence over time on the entire organism needs to be fully understood.

Ongoing research is traditionally published in scientific journals, including advances with respect to diseases, disease mechanisms and treatment opportunities [3]. Furthermore, various data in hospitals and other clinical settings, e.g. obtained in medical examinations, are documented as natural language reports. However, with the continuously increasing amount of available textual content, manually accessing these reports is a daunting task [4]. As a consequence, text mining and natural language processing tools have gained more and more importance to enable the identification of topic-relevant papers (information retrieval), e.g. GoPubMed [5] for the retrieval of Gene Ontology (GO) term relevant publications. Moreover, text mining tools can also help with the extraction of potentially relevant facts (information extraction), e.g. Protein Corral [6] that allows a user to extract protein interactions from abstracts of scientific publications. Some of the information extraction tools support the mapping to database identifiers or ontological concepts, such as the National Center for Biomedical Ontology (NCBO) annotator [7]. This process is referred to as *normalisation*, or *concept recognition* in the case of normalising to ontological concepts.

Several different methods are used to identify facts from textual data covering basic dictionary approaches as well as complex machine learning systems. With the differences in methods, we experience a variance in the adaptability to and the performance in a particular domain of each of the individual methods. In order to verify the different methods and provide a means of comparison, large text collections (‘corpora’) are needed that are annotated with respect to the information that is to be extracted by automated means. Examples for corpora include the Colorado Richly Annotated Full Text (CRAFT) corpus [8], the Penn Treebank corpus [9] and the GENIA corpus [10]. Manually created corpora are usually of relatively small size as they are expensive to generate, and specific to a domain of interest. As they constitute benchmark data, they are referred to as a gold standard (GS) annotation set.

Due to the time and costs involved in manually generating corpora, some studies lately investigated options to automatically annotate corpora with annotations, without intervention from a domain expert [4]. To distinguish between corpora that include human intervention and possess a perceived higher quality, automated corpora were coined ‘silver standard’ corpora. For example, the Collaborative Annotation of a Large Biomedical Corpus (CALBC) project [11] investigated solutions to automatically generate annotations for a subset of abstracts contained in Medline (https://www.nlm.nih.gov/bsd/pmresources.html) with entities that are relevant to the medical domain, e.g. drugs, and protein and gene names. While the project delivered an approach for the generation of silver standard annotations [12], the types of entities were limited and only abstracts of scientific publications were included in the analysis. Clinical documentation such as Electronic Health Records (EHR) were entirely neglected in the CALBC project.

Traditionally, most of the information retrieval and extraction solutions focused on gene names and products. Only fairly recently, solutions specialising on the extraction of phenotype data were developed [13]. Generalised concept recognition systems support the annotation of text with a multitude of ontologies, including phenotypes and other disease-related entities. However, due to the generality of the algorithms applied, the concept recognition systems may not be well-adapted to specific biological and biomedical domains. In earlier studies it has been shown that the existing generalised solutions are insufficient for specific annotation purposes [14]. In a similar study, Funk and authors showed that the performance of individual systems varies with the extracted concept type [15]. Thus, new specialised solutions are required that facilitate the targeted extraction of phenotypes and allow for peculiarities that influence the performance of phenotype extraction systems.

In order to build these specialised extraction methods, phenotype corpora are needed for evaluation purposes. Due to the lack of tools specialised in phenotype extraction, the amount of available gold standard data for evaluation purposes is small [13]. In this study, we leverage the annotations of four established concept recognition systems (the NCBO annotator, the clinical Text Analysis and Knowledge Extraction System (cTAKES – https://ctakes.apache.org/), the Biomedical Concept Annotation System (BeCAS) [16], and MetaMap [17]) for the generation of silver standard annotation sets on medical as well as biological data. In the silver standard annotation sets, we focus on phenotypes as wells as phenotype-related concepts such as diseases, with the ultimate aim to generate a high-quality phenotype corpus for the evaluation of specialised phenotype extraction systems. Our results show that when combining the annotations of the individual systems into a silver standard annotation set with the method derived in the CALBC project [12], the resulting annotation set is biased towards two systems (NCBO annotator and cTAKES). Comparing the obtained silver standard annotations to a gold standard shows a promising F1-measure of 33% but also indicates limitations that need to be overcome in order to automatically produce a high-quality annotation set. Furthermore, we investigated how each of the individual system influences the quality of the silver set annotations. From this work, we derived insights into the creation of silver standards for disease-related concept for future work. In conclusion, individual system performances as well as leveraging strategies for the generation of silver set annotations need to be revised for diseases-related concepts such as phenotypes. This study is a continuation from our previous work presented in [18].

The textual content of the PubMed corpus, accession numbers for the clinical trials corpus, assigned annotations of the four concept recognition systems as well as the generated silver standard annotation sets are available from http://purl.org/phenotype/resources or http://dx.doi.org/10.6084/m9.figshare.1257838. The textual content of the ShARe/CLEF (https://sites.google.com/site/shareclefehealth/data) and i2b2 (https://i2b2.org/NLP/DataSets/) corpora needs to be requested with the individual corpus providers.

## Results

### NCBO annotator and cTAKES perform best on silver standard corpora

**Figure 1:**
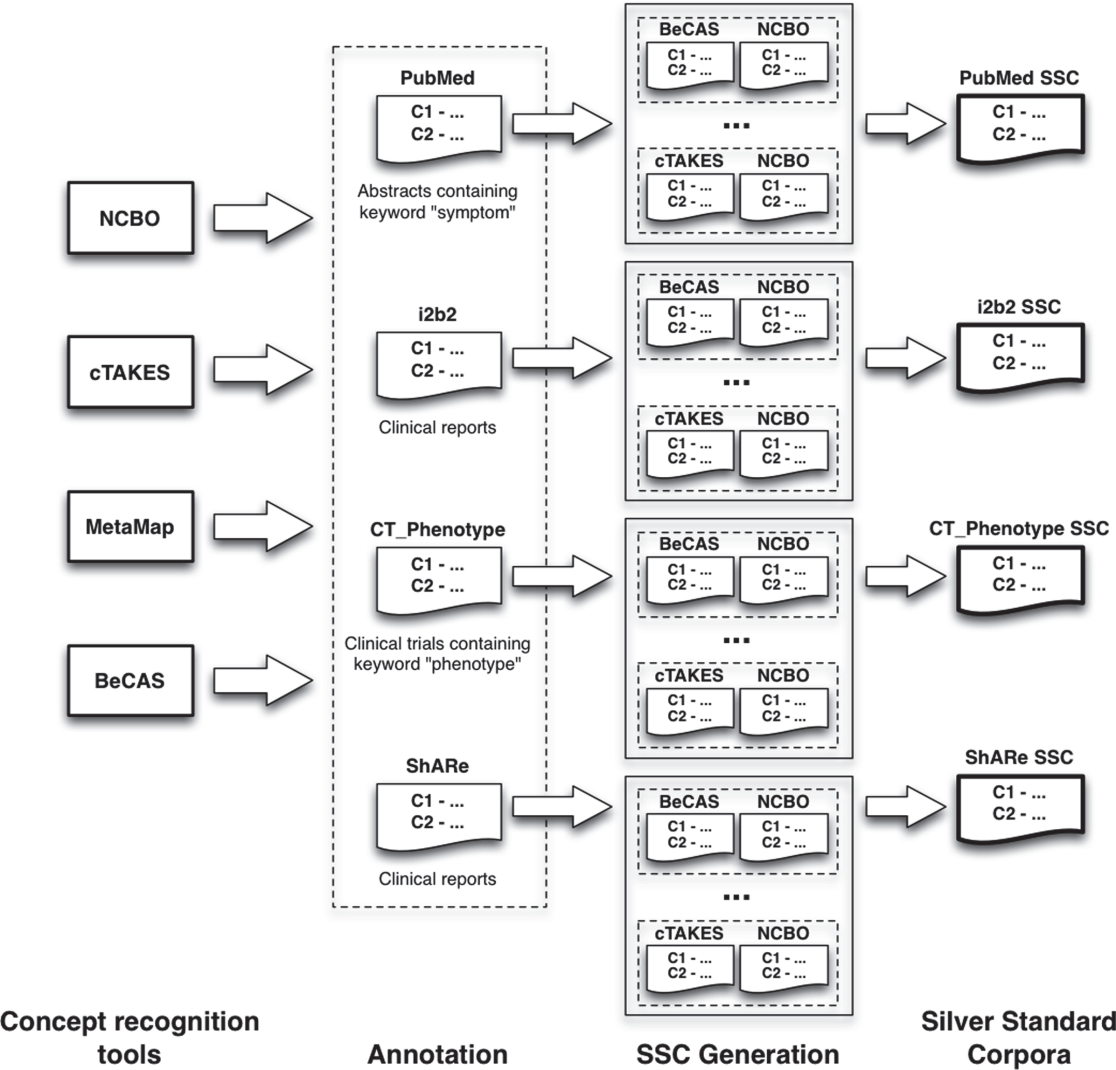
Generation of silver standard annotations depends on individual, independent concept recognition system annotations. Each system assigns annotations for each of the four corpora independently. After all the annotations are obtained, the systems’ outputs are harmonised on a corpus level. Two out of four systems have to agree on an annotation in order for it to be propagated to the silver annotation set.

In order to automatically generate silver standard corpora for four different textual resources, we used four established concept recognition systems and a standard method for integrating the annotations of the individual systems [12] (see Fig. 1 and section *Concept recognition systems* for more details). The output of each of the concept recognition tool was limited to the annotation of entities that can be represented using the concept unique identifiers (CUI)s from the Unified Medical Language System (UMLS) [19]. To evaluate the performance of the individual systems with respect to the four generated SSC, we measured precision, recall and the F1-measure based on an *exact* and a *sentence-level* matching. The obtained results are presented in Table 1.

**Table 1:** Silver standard evaluation.

		PubMed		i2b2		CT_Phenotype		ShARe (SSC)	
		exact	sent	exact	sent	exact	sent	exact	sent
NCBO	P	0.37	0.37	0.06	0.44	0.44	0.47	0.11	0.50
	R	0.98	0.91	0.81	0.86	0.96	0.87	1	0.93
	F1	**0.54**	**0.52**	**0.12**	**0.59**	**0.60**	**0.61**	**0.21**	**0.64**
cTAKES	P	0.62	0.65	0.05	0.58	0.68	0.68	0.23	0.61
	R	0.91	0.78	0.34	0.52	0.81	0.58	0.95	0.54
	F1	**0.74**	**0.71**	0.09	0.54	**0.74**	**0.62**	**0.37**	0.57
BeCAS	P	0.15	0.77	0.25	0.74	0.18	0.71	0.00	0.08
	R	0.02	0.10	0.02	0.01	0.03	0.09	0.00	0.01
	F1	0.04	0.18	0.03	0.01	0.05	0.15	0.00	0.02
MetaMap	P	0.23	0.51	0.10	0.50	0.24	0.50	0.00	0.58
	R	0.13	0.35	0.86	0.80	0.30	0.67	0.02	0.72
	F1	0.17	0.42	**0.18**	**0.62**	0.27	0.51	0.01	**0.64**

For each of the four corpora, a SSC was generated from the annotations of the four concept recognition systems. The performance of each of the systems was compared to each of the SSCs and precision, recall and harmonised F1-measure were calculated. In most cases, NCBO annotator and cTAKES perform as the best two systems out of the four applied.

Our results show that independent from the text type of the four different corpora, NCBO annotator is always among the two best performing systems (F1-measures in the range of 12–64%) and is mostly joined by cTAKES. Only in the case of the i2b2 corpus and the *sentence-level* assessment of the ShARe/CLEF data, MetaMap replaces cTAKES among the two best performing systems. In general, NCBO annotator achieves a high recall for all different types of text (81–100%) while the precision is lower (6% in the worst case). NCBO annotator’s high recall and lower precision in the silver standard evaluation indicates that NCBO annotator assigns more annotations per text span than the other three systems.

In addition to outperforming cTAKES in the case of the i2b2 corpus and the *sentence-level* evaluation on the ShARe/CLEF data, MetaMap in general shows better F1-measures in the *sentence-level* evaluation (42–64%) than in the case of an *exact* matching. This suggests that MetaMap annotates similar entities than cTAKES and NCBO annotator but the text spans that correspond to the annotation differ from the other two systems. MetaMap mostly outperforms NCBO annotator with regards to the precision of the annotations which suggests that MetaMap agrees with the annotations assigned but assigns on average a smaller set of annotations per text span than does NCBO annotator.

Despite the fact that BeCAS shows a high precision for the PubMed, i2b2, and CT_Phenotype corpus in a *sentence-level* evaluation (71–77%), the obtained F1-measures do not scale with the precision due to a low recall. The comparatively high precision could be seen as an indicator for BeCAS assigning less annotations to individual text spans than the other systems. The best F1-measure is achieved in the case of the *sentence-level* evaluation on the PubMed abstract corpus which suggests that the annotation procedure works in agreement with the other systems on abstracts but not on the other text types represented by different corpora applied here.

With the requirement that at least two systems have to agree on an annotation for it to be propagated to the silver standard annotation set (see *Generation of Silver Standard Corpora*), we can assume that the best performing two systems are those that influence the silver standard annotation sets the most. This is, however, no indication of the correctness of the annotations of the silver standard and only suggests closeness of systems in terms of annotation strategies. From our results it becomes clear that out of the four applied concept recognition systems, cTAKES and NCBO annotator share significant overlap in their annotations, and as a consequence, control the annotations propagated to the silver standard from the individual systems’ output.

### NCBO annotator and cTAKES perform best on gold standard

While the evaluation of the individual systems on the silver standard corpora shows the contribution of the system towards these corpora, it cannot be used as an indicator for the correctness of the annotations. In order to assess the correctness of the silver standard annotations, we included one corpus providing gold standard annotations; we employed the ShARe/CLEF data (annotations from 99 documents in the test set). The results obtained from these comparisons are provided in Table 2.

**Table 2:** Gold standard evaluation.

		ShARe_GS exact	ShARe_GS sentence
NCBO	P	0.02	0.04
	R	0.23	0.51
	F1	**0.03**	**0.07**
cTAKES	P	0.09	0.10
	R	0.60	0.63
	F1	**0.16**	**0.17**
BeCAS	P	0.00	0.01
	R	0.00	0.01
	F1	0.00	0.01
MetaMap	P	0.00	0.04
	R	0.00	0.35
	F1	0.00	0.07
SSC	P	0.15	0.18
	R	0.44	0.53
	F1	**0.23**	**0.27**

Evaluation of systems against gold standard and comparison between the ShARe/CLEF silver and gold standard.

Our results show that NCBO annotator and cTAKES perform best when evaluating each of the four systems relative to the ShARe/CLEF gold standard annotations, both on *exact-* and *sentence-level*. Among all four tested concept recognition systems, cTAKES has the overall best performance on the gold standard. This means that the high contributions from NCBO annotator and cTAKES towards the silver standard may be an advantage for the correctness of annotations. While MetaMap outperformed cTAKES on the ShARe/CLEF SSC (see last column, Table 1), it showed a lower performance on the gold standard (F1-measure of 7% at best). Both NCBO annotator and cTAKES show a strong recall in the evaluation, but the precision causes the F1-measures to be in the range of 3–17%. BeCAS showed a low overall performance which may be a further indicator that BeCAS does not include methodology to treat clinical report files appropriately.

When comparing the silver and gold standard annotations with precision, recall and F1-measure, we see that there is a discrepancy between both annotation sets. While the recall is higher, the precision of the silver set annotations only reaches 18% at best. The decreased precision causes the F1-measures to be in the range of 23–27%. In order to reduce the discrepancy of gold and silver annotation sets, ways need to be identified that allow the improvement of the precision of the silver standard annotations by reducing the amount of incorrectly assigned annotations as well as bringing in systems that allow for the annotation of concepts that cannot be covered with either of the applied systems.

### NCBO annotator and cTAKES are sufficient to achieve maximum quality of silver standard

To assess the contribution of the individual systems to the quality of the silver standard corpora, we executed a reduction experiment by means that the number of systems contributing to the SSC were reduced while the SSC creation rules were maintained. We included all possible combinations of the four concept recognition systems to see whether there is an ideal combination of any of the four concept recognition systems with respect to the quality of the obtained SSC that could have been masqueraded with the inclusion of another system. For example, removing a system that causes the inclusion of a large number of incorrect annotations by agreeing to incorrect annotations from other systems, can improve the quality of the silver standard. Table 3 provides the results of the reduction experiment.

**Table 3:** Contribution of individual systems to the quality of the silver standard corpora.

no sys	NCBO	cTAKES	BeCAS	MetaMap	P(E)	R(E)	F1(E)	P(S)	R(S)	F1(S)
4	y	y	y	y	0.15	**0.44**	**0.23**	0.18	**0.53**	0.27
3	y	y	y	n	0.16	0.43	**0.23**	0.26	0.44	**0.33**
	y	y	n	y	0.15	**0.44**	**0.23**	0.18	**0.53**	0.27
	y	n	y	y	0.04	0.01	0.01	0.15	0.26	0.19
	n	y	y	y	**0.22**	0.02	0.04	0.33	0.27	0.30
2	y	y	n	n	0.16	0.43	**0.23**	0.26	0.44	**0.33**
	y	n	y	n	0.00	0.00	0.00	0.27	0.01	0.02
	y	n	n	y	0.04	0.01	0.01	0.15	0.25	0.19
	n	y	y	n	0.00	0.00	0.00	0.31	0.01	0.02
	n	y	n	y	**0.22**	0.02	0.04	0.34	0.26	0.30
	n	n	y	y	0.00	0.00	0.00	**0.42**	0.01	0.01

When comparing the contribution of individual systems to the quality of the SSC, we see that the annotations of two systems (NCBO annotator and cTAKES) are the two systems contributing the most to the quality of the SSC. Adding MetaMap increases the recall but lowers precision, and in the case of a sentence-based comparison, the F1-measure.

As the results show, the best performance (F1-measure 33%) is achieved solely by combining NCBO Annotator’s and cTAKES’ annotation and evaluating on a *sentence-level*. While adding BeCAS did not change the overall performance, using MetaMap in conjunction with NCBO annotator and cTAKES reduced the F1-measure to 27%, and with that the quality of the silver set annotations. In the case of the *exact* evaluation, adding MetaMap and/or BeCAS does not change the F1-measure, which is consistently 23% for all four highest scoring combinations. This means that with the chosen settings for the individual systems and the silver standard propagation scheme, NCBO annotator in combination with cTAKES are sufficient to achieve the best possible quality of the silver set annotations.

The second best option (with an F1-measure of 30%) is the combination of cTAKES and MetaMap when evaluated on a *sentence-level*. *Exact* evaluation showed that the combination of both systems possessed, however, a low F1-measure of 4%. This drop in performance is an indicator that MetaMap assigns correct annotations with regards to the CUI and semantic type used for the annotation, but the text span within the sentence is incorrect with respect to the gold standard. While the combination of cTAKES with NCBO annotator leads to a higher recall (over precision), the combination of cTAKES and MetaMap leads to an improved precision (over recall).

Combining BeCAS and MetaMap solely achieves the highest possible precision of 42% when evaluated on a sentence level, the recall of this combination is at 1%. This result illustrates that both systems recover only a small subset of the gold standard annotations but the boundaries differ from those provided in the gold standard. An ideal combination of the four systems would enable to use the breadth of annotation provided by NCBO and cTAKES while using a refinement in precision from BeCAS and/or MetaMap on a *sentence-level*.

## Discussion

### Leveraging strategy needs adaption to phenotype-related domains

When integrating the annotations of the four concept recognition systems, we applied the rule that at least two of the four systems have to agree on an annotation (see section *Generation of Silver Standard Corpora*). Implied by this rule is that the systems that achieve the highest performance measures in the silver standard evaluation are likely to be the systems that contribute the most to the annotations added. When assessing the performance of the systems against the silver standard corpora, in three out of the four cases, NCBO annotator and cTAKES show the highest performance.

Out of the four systems, BeCAS shows the lowest performance on all four different SSC. This behaviour can be explained with BeCAS’s annotation policy that requires only the longest text span to be annotated. All the other systems allow for multiple, overlapping annotations with varying length, and may therefore be closer to one another in the content they are annotating. We also found indications that BeCAS converts anatomical adjectives to anatomic concepts; a behaviour the other systems do not exhibit to the best of our knowledge. Furthermore, the highest performance for BeCAS is achieved on the PubMed corpus which may indicate a bias towards abstract texts that are linguistically different from clinical documents. This behaviour may be an artifact of BeCAS’s performance being assessed on journal publication data instead of clinical documents [16].

In the case of the i2b2 corpus, the two best performing systems are MetaMap and NCBO annotator. This suggests a superiority of MetaMap on clinical records over cTAKES. MetaMap possesses built-in support for ambiguity, abbreviation recognition and sentence splitting that is geared towards the handling of medical text while the integrated support in cTAKES is limited. These differences in strength with respect to the type of text that is analysed may have to be taken into consideration when leveraging annotations from the individual systems’ output to the silver standard annotation set.

Using a reduction experiment shows that the best recall and F1-measure (44% and 33% respectively) of the SSC vs GS is achieved by combining annotations from NCBO Annotator and cTAKES. However, the best precision is achieved when using annotations from BeCAS and MetaMap. Adding BeCAS to the pool of annotations does not change the results while adding MetaMap causes a drop in performance. This drop in performance indicates that the leveraging strategy applied here is not ideal for the concept recognition systems and textual data applied.

In general the obtained results show that the composition of the SSC corpora varies with the matching techniques employed. If we allow a *sentence-level* matching technique for the inclusion of annotations, then systems such as MetaMap and BeCAS show an increased F1-measure which is due to the inclusion of more annotations that are identified by each of the two systems. This means that both systems assign annotations that are in agreement with the other systems but the boundaries are not harmonised across the different systems. Thus, a different leveraging strategy for SSC annotation than applied in this study is required.

### ShARe/CLEF data challenges concept recognition systems

When assessing the overall performance of the individual concept recognition systems on the gold standard annotation set, we see a different picture as compared to the performance measures on the silver set annotations. While MetaMap shows a good performance (64% F1-measure on a sentence level) on the silver set annotations, it shows an F1-measure of only 7% on the gold standard annotation set. This shift in performance highlights the limitations of the silver standard annotations for phenotype-related concepts. In a preliminary examination, we were able to distinguish different problematic areas causing the difference between gold and silver standard annotations.

**Boundaries:** Because each of the system is assigning annotations independent from the other systems and each system has its own algorithm to identify text spans corresponding to entities, the entity boundaries are not harmonised and can differ between the applied systems. Furthermore, as shown by the results provided in Table 2, all the performance measures for each of the systems improve when using a *sentence-level* evaluation as compared to the *exact* evaluation. If two systems agree on an annotation, even though the location is wrong, this annotation will be propagated to the silver standard and cause a decreased quality of the silver standard. From the results, it seems better to let systems vote on *sentence-level* instead of *exact* annotations but this observation needs further validation.

**Short forms of terms:** In addition to boundaries of entities, another source for missing and incorrect annotations in the silver standards, is the extensive usage of short forms in medical reports that are not further defined. For example, the text span *la enlargement* refers to the gold standard annotation *left atrium enlargement* (CUI:C0344720). Without a preprocessing step that would expand these abbreviations, each system would – if it possesses methodology – apply its own algorithm for term expansion, potentially leading to different results as compared to the other systems.

**Lack of context:** Further annotations are missed or incorrectly assigned due to the lack of context in the original text sources. The sentence *On motor exam, there is generally decreased bulk and tone, decreased symmetrically, there is generalized wasting […]* is annotated with *muscle wasting* (CUI:C0026846; based on *motor* and *generalized wasting*) and *decreased muscle tone* (CUI:C0026827; based on *motor* and *decreased tone*). Due to the expression being split into several parts and scattered across the sentence, concept recognition tools fail to assign the correct annotations.

**Coordination of terms:** An additional cause for missing and incorrect annotations in the SSC is the case of the coordination of terms. Terms are coordinated using lists and connectors such as *and* or *or*. A coordinated term such as *abdomen soft, non-tender, non-distended* refers to two separate annotations in the gold standard: *abdomen soft* (CUI:C0426663) and *abdomen non-distended* (CUI:C0424826). Term coordination is a known issue that has been addressed in existing entity tagging pipelines but none of the systems employed here deals well with the issue. This issue could be solved with adding additional preprocessing before annotating the text sources and building a silver standard.

**Ambiguity/lack of context-dependent synonyms:** The last area for incorrect and missing annotations is the ambiguity of terms as well as the lack of context-dependent synonyms. A text span such as *heroin abuse* is annotated with *heroin dependence/addiction* (CUI:C0019337) in the gold standard. Neither of the systems was able to infer this annotation from the provided text and context in the original text source.

Some of these problematic areas can be addressed in future work by adding appropriate pre-processing methodology into the SSC creation pipeline, e.g. resolving short forms or dealing with coordination. For other areas, however, more work is required still to find adequate solutions, especially the lack of context in clinical documentation as well as the complex nature of phenotype descriptions.

In an earlier study [20], Rebholz-Schuhmann and colleagues investigated the correspondence between tagging solutions for protein and gene names and gold standard corpora. The authors observed a better performance of all the taggers on the two most recently developed corpora and speculated that this could be explained with the advancement of standardised naming conventions. Due to a later recognition of the importance of phenotypes, naming conventions have not been established yet, and little harmonisation between clinical and biological descriptions has happened. This also means that the performance of individual concept recognition systems as well as their combination to automatically generate corpora can be improved by defining naming conventions that are harmonised across the biomedical domain.

### Conclusions

Using four established concept recognition systems and a standard method to build a SSC for phenotype-related concepts reaches in the best case a F1-measure of 33% if only NCBO annotator and cTAKES are used for annotations. Even if four systems are combined, the obtained SSC is limited to mostly annotations of these two concept recognition systems. However, the best precision is achieved when combining MetaMap and BeCAS. While performance can be improved by adding pre-processing steps to the overall pipeline, the performance of the individual concept recognition systems needs to be improved. More consistent annotations across the four systems are required in order to be able to harvest these annotations for an automatically generated, high quality reference corpus for phenotype and disease-related concepts. Furthermore, the leveraging strategy for the output from the individual systems needs to be revised and adapted with respect to the domain of phenotypes and disease-related concepts.

## Materials and Methods

### Corpora

In this study, we used four different corpora (further explained in the following paragraphs) to build and assess the quality of an SSC generated by the application of four concept recognition systems and a standard leveraging strategy [12]. As individual systems can perform differently depending on the text type and phenotype recognition systems are needed for scientific literature as well as free text in patient records, we aimed to cover a variety of text types. Instead of limiting ourselves to abstracts from PubMed, we also used clinical reports and trials.

To represent phenotypes and disease-related concepts in each of the corpora, we used the UMLS. The UMLS Metathesaurus forms the core of the UMLS and incorporates over 100 source vocabularies including the NCBI taxonomy [21] and SNOMED CT. UMLS provides an identifier (CUI) as well as a semantic type for each individual medical concept. The UMLS Metathesaurus includes 135 semantic types, e.g. *Anatomical Abnormality* and *Sign or Symptom*, which are further summarised into semantic groups. In this study, we focus on CUIs and semantic types for the creation of the SSCs and the different evaluation strategies.

To evaluate the quality of the obtained SSC and provide a reference for the assessment as to which combination of systems achieves the highest quality of a SSC, we used the ShARe/CLEF corpus. The ShARe/CLEF corpus provides a gold standard annotation set based on UMLS’ CUIs.

**i2b2:**

The i2b2 corpus (https://i2b2.org/NLP/DataSets) consists of 693 clinical reports, made available under a restricted license by Partners HealthCare. The experiments described in this paper have been carried out on the complete 2010 Relations Challenge corpus (i.e., both training and testing datasets). Reports consist of a series of section headings such as *HISTORY OF PRESENT ILLNESS*, *ALLERGIES*, followed by the actual description. The description ranges from a set of bullet points such as previous medication, to short descriptive enumerations such as findings at the clinical investigation, and finally, to longer free form phrases which are usually explanatory notes on the hospital course or the social history. This aspect is particularly important, because it may raise segmentation as well as tokenization challenges for any tool that relies on sentence splitting as a pre-processing step. We notice also that the length of the i2b2 texts is characterised by significant variation from the mean of 904 tokens.

**PubMed corpus:**

Our PubMed corpus consists of 2,163 Medline abstracts and was retrieved by querying PubMed for the keyword *symptom*. As a remark, we have used *symptom* instead of *phenotype* because we have assumed that the resulting publications will have to some extent a more clinically-oriented language, i.e., a language similar to the one used in i2b2. From a statistical perspective, as opposed to the i2b2 corpus, we can observe that word tokens have a much higher degree of duplication (1.6 versus 1.02). The overlap between the two corpora is 8,818 unique tokens, i.e., 24.69% of the PubMed corpus and 28.72% of the i2b2 corpus.

**Clinical trials corpus:**

The clinical trials corpus consists of clinical trials downloaded from http://clinicaltrials.gov/ using the keyword *phenotype*, since *symptom* would be too common in this collection. An interesting finding of the clinical trials corpus is its large token/uniqueness ratio of 12.94, compared to 1.60 in PubMed. This entails a high degree of repetition and a fairly restricted and uniform vocabulary of the language used within them. The overlap between the two corpora in terms of unique tokens is around 30%, i.e. 11,216 tokens.

**ShARe/CLEF corpus:**

As a forth text corpus, we chose to use the ShARE/CLEF e-health 2013 Task 1 evaluation data set (https://sites.google.com/site/shareclefehealth/data) of 300 de-identified clinical records from the MIMIC II database with stand-off annotations for disorders. This is a mixed corpus that includes discharge summaries, echo reports and radiology reports used in an intensive care unit setting. 200 notes were designated for training and 100 for testing. Annotation was done by two annotators plus open adjudication. Access to the corpus required appropriate registration with MIMIC II and the completion of a US human subjects training certificate. In this study, we used only the 100 training documents, out of which one of the documents was empty. As can be seen in Table 4, the ShARe/CLEF corpus possesses a high number of entities with respect to its size, a medium token/uniqueness ratio compared with the other corpora, and a high density of entities per document.

**Table 4:** Corpora statistics.

	PubMed	i2b2	CT_Phenotype	ShARe
No. documents	2,163	693	906	99
Total token count	57,414	31,526	503,367	86,599
Unique token count	35,706	30,699	38,898	13,742
Token/uniqueness ratio	1.60	1.02	12.94	6.30
Avg. token count/doc	279.09	904.27	555.59	874.73
Token Std. dev.	67.49	616.12	428.62	470.71
Disease domain	mixed	mixed	mixed	mixed
Text type	abstracts	clinical reports	clinical trials	clinical reports

Statistics for the four different corpora employed in this study. While the PubMed corpus possesses the highest number of documents, the CT_Phenotype corpus contains the most unique tokens.

Out of the four corpora applied here, this corpus is the only one that possesses a gold standard annotation set. The gold standard annotation set includes 7,076 annotations in total: 5,351 that are mapped to a CUI (796 unique CUIs in total), 1,723 that cannot be mapped to a UMLS CUI, and two that cannot be found in the text. The 5,351 CUIs fall into 15 semantic groups (highly represented groups are *Disease or syndrome* and *Sign or Symptom*), but five groups are only represented with one concept.

### Concept recognition systems

To generate the different SSC, we applied four established concept recognition systems, each of which is described in the following. Our choice of systems was aimed at a variety of tools that were developed with different goals in mind (to span with the variety of text types): NCBO annotator being a universal concept recognition tool for a broad range of ontologies, cTakes developed for the analysis of electronic health records using SNOMED CT and the RxNORM subset of UMLS, BeCAS designed to analyse the scientific literature for a number of concept types such as diseases and chemical entities, and MetaMap to annotate any text with concepts from the UMLS.

**NCBO annotator:**

The NCBO annotator (http://bioportal.bioontology.org/annotator) is an online system that identifies and indexes biomedical concepts in unstructured text by exploiting a range of over 300 ontologies in the UMLS and NCBO BioPortal [22]. These ontologies include many that have particular relevance to disorders and phenotypes such as SNOMED CT [23], LOINC [24] and the Foundational Model of Anatomy [25].

NCBO annotator operates in two stages: concept recognition and semantic expansion. Concept recognition performs lexical matching by pooling terms and their synonyms from across the ontologies and then applying a multiline version of grep to match lexical variants in free text. During semantic expansion, various rules such as transitive closure and semantic mapping using the UMLS Metathesaurus are used to suggest related concepts from within and across ontologies based on extant relationships. The mappings and the depth of transitive closure are customisable within the tool.

**cTAKES:**

cTAKES from Mayo Clinic consists of a staged pipeline of modules that are both statistical and rule-based. The order of processing is somewhat similar to MetaMap and consists of the following stages: sentence boundary detection with OpenNLP (https://wiki.apache.org/solr/OpenNLP), tokenization, lexical normalisation (SPECIALIST lexical tools), POS tagging and shallow parsing using OpenNLP trained in-domain on Mayo Clinic EHRs, concept recognition, negation detection using NegEx [26] and temporal status detection. Concept recognition is conducted within the boundaries of noun phrases using dictionary matching on a synonym-extended version of SNOMED CT and RxNORM [27] subset of UMLS. cTAKES was subject to a rigorous component-by-component evaluation during development. During this process, although the focus of testing was on EHRs, the system was also tested on combinations of the GENIA corpus of Medline abstracts and Penn Treebank corpus.

**BeCAS:**

BeCAS (http://bioinformatics.ua.pt/becas) [16] from the University of Aveiro is the newest integrated system of the four that we tried. The pipeline of processes involves the following stages: sentence boundary detection, tokenization, lemmatization, part of speech tagging and chunking, abbreviation disambiguation, and CUI tagging. The first four stages are performed by GDep [28], a dependency parser that incorporates domain adaptation using unlabelled data from the target domain. CUI tagging is conducted using regular expressions for specific types such as anatomical entities and diseases. Dictionaries used as sources for the regular expressions include the UMLS, LexEBI [29] and the Jochem joint chemical dictionary [30]. During development the concept recognition system was tested on abstracts and full length scientific articles using an overlapping matching strategy.

**MetaMap:**

MetaMap (http://metamap.nlm.nih.gov/) [17] is a widely used system from the NLM for finding mentions of clinical terms based on CUI mappings to the UMLS Metathesaurus. The system exploits a fusion of linguistic and statistical methods in a staged analysis pipeline. The first stages of processing perform mundane but important tasks such as sentence boundary detection, tokenization, acronym/abbreviation identification and POS tagging. In the next stages, candidate phrases are identified by dictionary lookup in the SPECIALIST lexicon [31] and shallow parsing using the SPECIALIST parser [32]. String matching then takes place on the UMLS Metathesaurus before candidates are mapped to the UMLS and compared for the amount of variation. A final stage of word sense disambiguation uses local contextual and domain-sensitive clues to arrive at the correct CUI. MetaMap is highly configurable, for example, users have the option to specify their own vocabulary lists (e.g. for abbreviations), use negation detection and the degree of variation between text mention and UMLS terms.

### Generation of Silver Standard Corpora

The annotation process described in the previous section resulted in an individual corpus-specific set of annotations produced by each of the four systems. In order to create silver standard versions of our four corpora, we used two matching techniques (*sentence-level* and *exact*) and one inclusion rule. The inclusion rule determines the annotations that will be included in the silver standard based on a given matching technique. Rebholz-Schuhmann et al. [12] performed experiments with several such inclusion rules and the best results have been achieved by including all annotations matched by at least two systems. We have generated four silver standard corpora (one for each of the in this study utilised corpora) using the same rule, i.e., for all annotations produced by the four systems in the context of each entry in a particular corpus, if an annotation is matched by at least two systems, it is subsequently included in the silver standard for this corpus.

**Figure 2:**
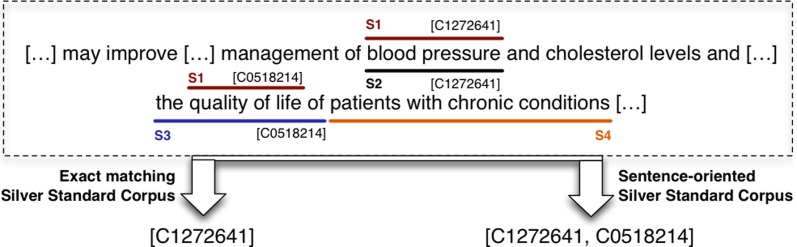
Example sentence illustrating the annotations obtained from the four concept recognition systems and propagation to silver standard corpus. Each of the four concept recognition systems assigns annotations to the textual content of the corpora independent from the other systems. If two systems annotate the same text span, e.g. *blood pressure* (CUI:C1272641) or *quality of life* (CUI:0518214) in the case of a sentence-based matching, this text annotation is included into the silver standard corpus of the text source.

The second component of this generation process is the matching technique, which determines the degree of alignment between a pair of annotations. In our study, we used two different matching techniques: *exact* and based on a *sentence-level*. Let us consider the example depicted in Fig. 2, including the annotations produced by NCBO annotator(*blood pressure* and *quality of life*), cTAKES (*blood pressure*), BeCAS (*the quality of life of*) and MetaMap (*patients with chronic conditions*). For brevity, we have not included the annotated CUIs. An ideal situation would lead to an exact match between the annotations produced by a pair of systems – for example, *blood pressure* annotated by NCBO annotator and cTAKES. Consequently, *blood pressure* would be included within the silver standard based on either *exact* or a *sentence-level* matching, while *quality of life* is included in the *sentence-level* SSC in addition.

### Assessment of system performances against Silver and Gold Standard Corpora

**Figure 3:**
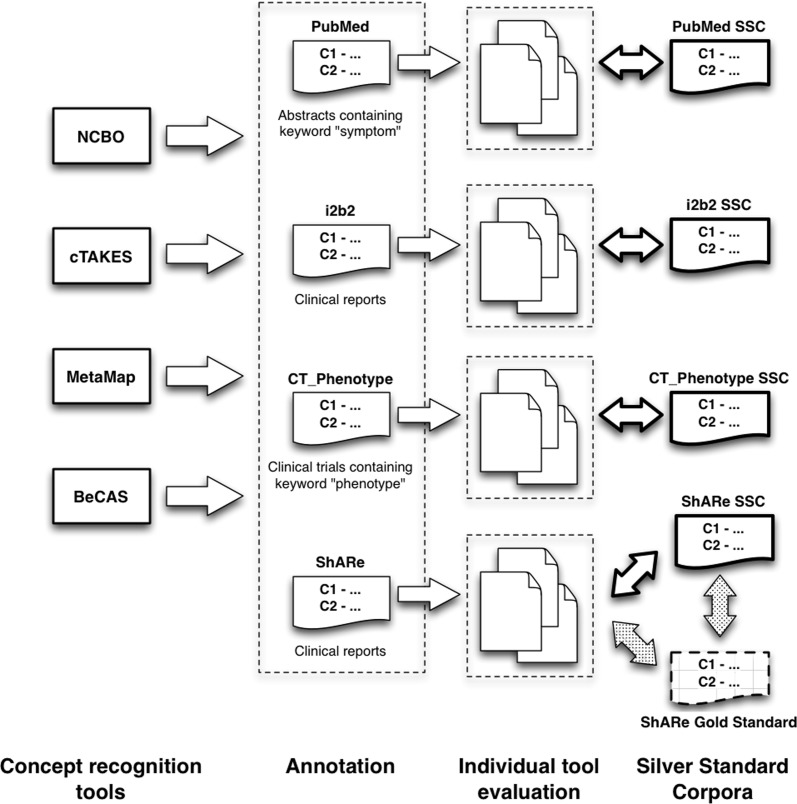
Each system is evaluated against each of the four generated silver standard corpora and, in addition, against the gold annotations of the ShARe/CLEF corpus. Concept recognition systems were evaluated by obtaining precision, recall and F1-measure based on the different applied annotation sets in this study. Each of the double-pointed arrows symbolises both types of evaluation: an *exact* and a *sentence-based*.

The performance measures were calculated with two different matching techniques for the location of the entities: *exact* and *sentence-level*. In the case of the *exact* comparison, an entity was considered to be a true positive (TP) if the CUI provided by the system is identical with the silver standard annotation and, at the same time, falls into exactly the same location. If an annotation does not match both criteria, it is assumed to be a false positive (FP). Due to the systems varying in text spans, we also introduced a more relaxed evaluation (*sentence-level*) by means that any annotation that possesses the correct CUI and falls into the same sentence as the one contained in the silver standard, is counted as a true positive (TP) while all other annotations are regarded as false positives (FP). To assess the performance of the individual systems, we calculated the precision P $\;(P=\frac{TP}{TP+FP})$, the recall R $\left(R=\frac{TP}{TP+FN}\right)$ and the harmonised f-measure F1 $\left(F1=\frac{2*precision*recall}{precision+recall}\right)$ for each of the systems individually. Fig. 3 illustrates the different evaluation strategies.

### Comparison against gold standard of ShARe/CLEF data

For the ShARe/CLEF corpus, a gold standard annotation set was available, allowing the assessment of the quality of the generated SSC as well as the performance of the individual systems. Even though we could not compare the other three silver standard corpora to a gold annotation set, the comparison of the silver standard to the gold standard can highlight general problems with the annotation and combination strategies applied here that are transferable across corpora.

We compared the performance of the four different systems and the quality of the silver standard annotations by calculation precision, recall and the harmonised f-measure. The evaluation procedure was kept identical to the evaluation of the individual systems against the silver standard to allow for comparability of the different results.

### Reduction experiment to assess contribution of systems to quality of SSCs

To assess the contribution of the individual concept recognition systems to the quality of the resulting silver standard corpora, we conducted a reduction experiment on the ShARe/CLEF data. The experiment was set-up to incrementally lower the number of contributing systems and assess all possible combinations of systems. The number of systems included varied from two to four. The overall voting rules for the generating of the SSC were maintained even though the number of systems was reduced. Each obtained SSC was then compared to the GS annotation set to determine the most valuable combination of systems with respect to generating a GS with the applied inclusion rule and matching techniques.

The performance measures calculated were, as in previous settings, precision, recall and F1-measure. We calculated these measures on an *exact* as well as *sentence-level* basis. In the case of the *exact* measurements (designated P(E), R(E), and F(E)), the CUI of the GS has to be matched in exactly the same text span by the SSC. In the case of the *sentence-level* matching, the entity CUI provided by the GS needs to be contained within the annotation set of the corresponding sentence of the SSC.
